# A Randomized Controlled Evaluation of the Efficacy of an Ankle-Foot Cast on Walking Recovery Early After Stroke: SWIFT Cast Trial

**DOI:** 10.1177/1545968315583724

**Published:** 2016-01

**Authors:** Valerie M. Pomeroy, Philip Rowe, Allan Clark, Andrew Walker, Andrew Kerr, Elizabeth Chandler, Mark Barber, Jean-Claude Baron, On Behalf of the SWIFT Cast Investigators, Lindsay Anderson

**Affiliations:** 1 University of East Anglia, Norwich, UK; 2 University of Strathclyde, Glasgow, UK; 3 University of Leeds, UK; 4 Stroke Managed Clinical Network NHS Lanarkshire, Airdrie, UK; 5 University of Cambridge and INSERM U894, Hopital Sainte-Anne, Sorbonne Paris Cité, Paris, France

**Keywords:** rehabilitation, stroke, orthotics, physical therapy, walking

## Abstract

*Background*. Timely provision of an ankle-foot orthosis (AFO) orthotist customized for individuals early after stroke can be problematic. *Objective*. To evaluate the efficacy of a therapist-made AFO (SWIFT Cast) for walking recovery. *Methods*. This was a randomized controlled, observer-blind trial. Participants (n = 105) were recruited 3 to 42 days poststroke. All received conventional physical therapy (CPT) that included use of “off-the-shelf” and orthotist-made AFOs. People allocated to the experimental group also received a SWIFT Cast for up to 6 weeks. Measures were undertaken before randomization, 6 weeks thereafter (outcome), and at 6 months after stroke (follow-up). The primary measure was walking speed. Clinical efficacy evaluation used analysis of covariance. *Results*. Use of a SWIFT Cast during CPT sessions was significantly higher (*P* < .001) for the SWIFT Cast (55%) than the CPT group (3%). The CPT group used an AFO in 26% of CPT sessions, compared with 11% for the SWIFT Cast group (*P* = .005). At outcome, walking speed was 0.42 (standard deviation [SD] = 0.37) m/s for the CPT group and 0.32 (SD = 0.34) m/s for the SWIFT Cast group. Follow-up walking speed was 0.53 (SD = 0.38) m/s for the CPT group and 0.43 (0.34) m/s for the SWIFT Cast group. Differences, after accounting for minimization factors, were insignificant at outcome (*P* = .345) and follow-up (*P* = .360). *Conclusion and implications*. SWIFT Cast did not enhance the benefit of CPT, but the control group had greater use of another AFO. However, SWIFT Cast remains a clinical option because it is low cost and custom-made by therapists who can readily adapt it during the rehabilitation period.

## Introduction

At discharge from rehabilitation, stroke survivors may only walk at 0.55 m/s, well below normal (1.2-1.4 m/s) and not even fast enough to cross a road before the pedestrian crossing lights change (0.8 m/s).^1^ Consequently, improving walking recovery is an important goal.^2^ Clinical guidelines recommend repetitive functional task training (eg, Donaldson et al^3^), but if stroke survivors have substantial weakness, such training presents a challenge.

An ankle-foot orthosis (AFO) positions the foot in relation to the lower leg to optimize normal alignment during gait and thus improve walking performance.^4^ The optimal type of AFO is considered to be a device customized for individuals by an orthotist.^4^ Obtaining this within an appropriate timescale early after stroke can be problematic in clinical practice.^4^ The device evaluated here was a SWIFT Cast, custom-made and fitted by a therapist within 24 hours. This trial was focused on the potential use of a SWIFT Cast to provide optimal alignment of the lower limb to the ground during walking. The specific aim was to begin testing the hypothesis that the use of a SWIFT Cast, provided as an adjunct to conventional physical therapy (CPT), enhances walking recovery early after stroke more than CPT alone. This trial embedded mechanistic investigation of (*a*) the biomechanical correlates of walking improvement in response to the 2 forms of therapy and (*b*) the potential use of baseline biomechanical characteristics and site of stroke lesion as prognostic indicators of response. Here we report the clinical efficacy investigation.^5^ The investigation of the potential indicators of beneficial response and the underlying mechanisms of response to both experimental and control therapies will be communicated in additional reports.

## Methods

### Design

This study was a randomized, controlled, observer-blind phase II trial. All outcome measures were evaluated at the end of the 6-week intervention phase and at 6 months after stroke. Participants did not wear the SWIFT Cast while measures were evaluated. Into this clinical trial were embedded investigations of (*a*) the biomechanical correlates of walking improvement in response to therapy and (*b*) potential use of baseline biomechanical characteristics and site of stroke lesion as prognostic indicators of response. The embedded biomechanical investigations used the same gait parameters used for the clinical efficacy investigation reported here. The embedded stroke lesion site investigation involved structural neuroimaging undertaken 3 to 8 weeks after stroke onset. These embedded investigations are to be reported in subsequent articles. The full protocol is as described in an earlier publication.^5^

### Setting and Participants

Recruitment was from 2 stroke rehabilitation services. Participants were 18+ years old; 3 to 42 days poststroke (infarct or hemorrhage); fit for rehabilitation; able to take 3 steps while supported by 2 people, but with (*a*) an abnormal initial floor contact and/or (*b*) impaired ability to take full body weight through the paretic lower limb in stance; had no contractures in, or loss of skin integrity over, the lower limb; and able to follow a 1-stage command.

### Randomization

Group allocation was ordered pretrial by an independent statistician. Minimization was used with (*a*) ability to walk independently, as assessed by the Functional Ambulation Category (FAC; higher = 3-5 and lower = 2 or less)^6^; (*b*) clinical assessment of primary motor cortex involvement in the stroke lesion (yes/no); and (*c*) clinical center (A/B). An independent telephone randomization service concealed group allocation until after baseline measures.

### Interventions

All participants received CPT (treatment as usual) that included interventions designed to enhance movement performance and functional ability (supplementary material). The experimental group also received a customized SWIFT Cast (supplementary material). During CPT, the SWIFT Cast was worn for walking retraining, and participants were asked to wear the SWIFT Cast for the whole of their waking day initially. As gait improved, daily use was adjusted appropriately.

Splinting techniques, including AFOs, were used in both clinical centers.^3^ There were clinical concerns that if AFOs were not used in CPT, therapy would be suboptimal. The verbal agreement for this trial with the clinical therapists was that they would maintain their pretrial practice but that individuals allocated to the SWIFT Cast group would not receive another AFO during the intervention phase between baseline and outcome measurement time points (6 weeks). Research and clinical therapists met regularly and as needed throughout the trial to consider this. In addition, there were regular trial management meetings involving the research therapists, principle investigators, trial manager, and chief investigator. Influencing the routine interventions (CPT) provided by the clinical team was neither possible nor desirable. There are ethical and research governance frameworks that are designed to protect people from undue influence before and during their involvement in a trial.

### Outcome Measures

The primary outcome measure was walking speed. Secondary outcome measures were the following: FAC,^6^ Modified Rivermead Mobility Index (MRMI),^7^ peak angular velocity of the knee,^8^ gait symmetry, and angle of the tibia with the ground during walking. Participants did not wear either a SWIFT Cast or AFO during conduction of measures. The rationale was that a SWIFT Cast was designed to enhance recovery of motor control for walking and not to compensate for its loss.

### Sample Size

It was estimated that with a sample size of 110, the trial had 80% power at 5% significance to detect a clinical improvement of 0.13 m/s for walking speed with a standard deviation (SD) of improvement of 0.24 m/s. With this sample size, a clinical improvement of 1.1 points on the FAC, assuming a SD of 2 points, could be detected. To allow for an attrition rate of approximately 10%, the target sample was 120 participants (60 in each group).

### Analyses

All participants were analyzed according to the group to which they were randomly allocated. The clinical efficacy analysis was carried out using analysis of covariance adjusting for the baseline values and any imbalance in factors between the 2 groups. When the assumptions of analysis of covariance were not met, a *P* value and 95% confidence interval were estimated using a nonparametric bootstrap with 10 000 repetitions. All results were checked for sensitivity to missing data by imputing the data using iterative chain equations.

The total amount of CPT received was compared between the 2 treatment groups. To account for the inherent nonindependence of therapy sessions on the same individual, a random-effects model was used, with participant as the random effect. Fixed effects were the factors used to stratify randomization.

### Trial Oversight

A favorable ethical opinion was obtained from the National Research Ethics Service (reference 09/H0310/87). A Trial Steering Committee and a Data Monitoring Committee with independent chairs were convened. The Trial Steering Committee met 6 times and the Data Monitoring Committee 4 times to ensure good conduct of the trial and safety of participants and to monitor data collection. A Trial Management Group, including public and patient representatives, met regularly to monitor day-to-day running of the trial.

### Serious Adverse Events

Serious adverse events were recorded from baseline to follow-up.

## Results

### Participant Flow

**Figure 1.**
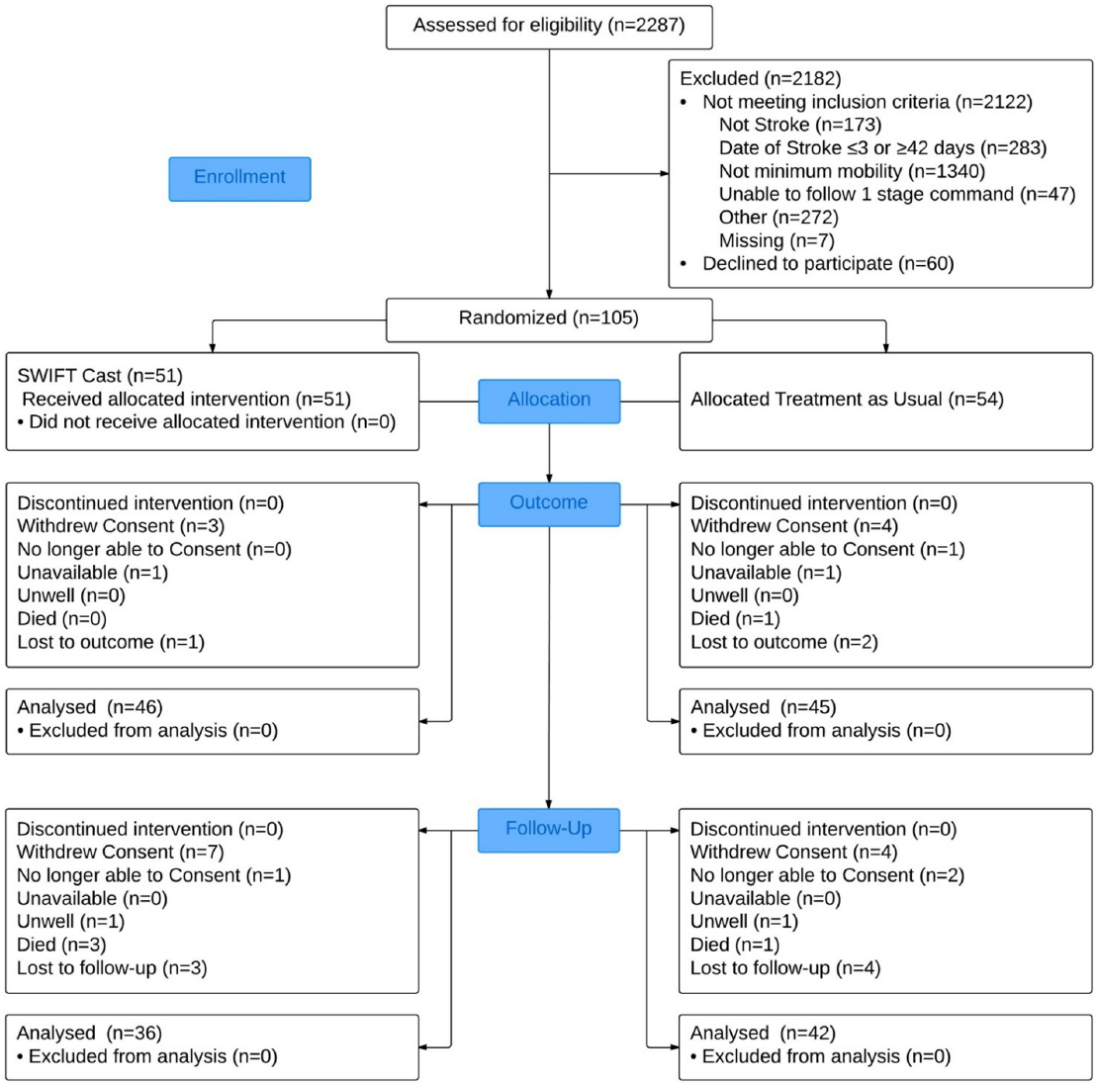
CONSORT flowchart.

Between October 20, 2010, and December 6, 2012, 2287 stroke survivors were screened (Figure 1). Of these, 2122 were ineligible, and 60 declined. Accordingly, 105 participants underwent baseline assessments and then were allocated randomly to either CPT (n = 54) or SWIFT Cast (n = 51). Measures postintervention (outcome) were completed by 46 (90.2%) of the CPT and 45 (83.3%) of the SWIFT Cast group. At follow-up, the measures were completed by 42 (77.8%) CPT and 36 (82.4%) SWIFT Cast participants. Reasons for attrition are shown in Figure 1.

### Baseline Characteristics

Baseline characteristics were balanced across the groups (Table 1). In summary, participants were a mean (SD) of 20.9 (19.0) days after stroke, with a mean age 66.7 (14.5) years. The right side of the body was more paretic for 50.4% of participants. For both groups, the mean walking speed was 0.1 (SD = 0.2) m/s and median FAC was 1.

**Table 1.** 

	Group	
Participant Characteristics at Baseline.^a^	CPT (n = 54)	SWIFT Cast (n = 51)
Age (years)	64.2 (15.3)	69.1 (13.6)
Time since stroke (days)	19.5 (11.3)	22.2 (26.7)
Lanarkshire site	33 (61.1)	31 (60.8)
Male	37 (68.5)	26 (51.0)
Type of stroke		
• Hemorrhage	8 (14.8)	8 (15.7)
• Infarct	45 (83.3)	41 (80.4)
• Missing	1 (1.9)	2 (3.9)
PMC involved in lesion	27 (50.0)	24 (47.1)
Right paretic side	29 (53.7)	24 (47.1)
FAC high categorization	15 (27.8)	10 (19.6)
FAC median	1 (0-3)	1 (0-2)
Walking speed (m/s)	0.1 (0.2)	0.1 (0.2)
MRMI total score	25 (8.4)	23.4 (7.5)
Peak knee velocity: nonparetic (degrees/s)	5.6 (10.2)	4.6 (9.4)
Peak knee velocity: paretic (degrees/s)	4.5 (8.4)	3.8 (8.2)
Able to walk 3 m independently		
• No	40 (74.1)	40 (78.4)
• Yes	13 (24.1)	10 (19.6)
• Missing	1 (1.9)	1 (2.0)
Gait parameters (n = 23)		
Tibial angle to vertical (degrees) at		
• Initial contact	−0.9 (8.8)	−1.6 (8.7)
• Foot flat	3.2 (6.0)	4.4 (6.3)
• Midstance	10.6 (5.1)	10.6 (3.7)
• Heel rise	28.8 (3.9)	28.3 (4.5)
• Terminal contact	40.7 (5.2)	40.8 (5.4)
• Midswing	26.1 (7.0)	28.2 (4.3)
Ratio of stance times	−0.1 (0.2)	−0.1 (0.2)
Ratio of step lengths	0.0 (0.1)	0.0 (0.1)
Ratio of peak angular velocities	−0.1 (0.1)	0.0 (0.1)

Abbreviations: CPT, conventional physical therapy; FAC, Functional Ambulation Category; MRMI, Modified Rivermead Mobility Index; SD, standard deviation; IQR, interquartile range; PMC, primary motor cortex.

a Values are mean (SD), median (IQR), or n (%).

### Routine CPT Received

In total, 895 sessions of CPT were provided (Table 2 and supplementary material; 487 for SWIFT Cast and 408 for CPT). The median (IQR) number of CPT sessions was 10 (0-16) for the SWIFT Cast and 7 (0-12) for the CPT group (*P* = .952). There were also no differences between the groups for the total duration of sessions (minutes) with median (IQR) values of 235 (0-590) and 140 (0-350) for the SWIFT Cast and CPT groups respectively (*P* = .792).

**Table 2.** Conventional Physical Therapy (CPT) Received by Both Groups and Use of Splinting Techniques in Centers.

Between-Group Comparison	CPT (n = 54)	SWIFT (n = 51)	Odds Ratio (95% CIs)	*P* Value
Number of sessions				
Total	408	487		
Median (IQR) per participant	7 (0.12)	10 (0.16)		.0952
Time duration (minutes)				
Total duration, median (IQR)	140 (0-350)	235 (0-590)		.792
Mean (SD) duration per participant session	33.23 (15.54)	39.06 (16.44)		.289^a^
Splinting techniques used				
Number (percentage of total) of sessions involving splinting techniques	313 (64.9%)	125 (31.4%)	0.06 (0.02, 0.22)	<.001
Strapping, number (%) of sessions	13 (3.3%)	5 (1.0%)	6.53 (0.40, 106.60)^b^	.188
Ankle foot orthosis (AFO), number (%)	104 (26.1%)	55 (11.4%)	10.36 (1.99, 53.95)	.005
SWIFT Cast, number (%)	10 (2.6%)	264 (54.9%)	0.00 (0.00, 0.02)	<.001
Participants used AFO, at least 1 session, number (%)	19 (35.2%)	9 (17.6%)	0.31 (0.12,0.83)	.020
Participants used SWIFT Cast, at least 1 session, number (%)	7 (13%)	32 (62.8%)	12.64 (4.49, 35.58)	
Between-center comparison of sessions	Centre A	Centre B		
Any splinting techniques, number (%)	272 of 538	166 of 342	0.89 (0.18, 4.36)	.887
Use of an AFO, number (%)	92 of 538	67 of 342	3.03 (0.39, 23.31)^b^	.288
	(17.1%)	(19.6%)		

Abbreviations: IQR, interquartile range; SD, standard deviation.

a Estimated from a random-effect linear regression model with participant as the random effect adjusting for factors used to stratify the randomization. To account for the nonnormality of the residuals a nonparametric bootstrap, with clustering variable as the participants, with 1000 repetitions, was used to estimate the *P* value and confidence interval.

b Estimated from a random-effect logistic regression model with participant as the random effect adjusting for factors used to stratify the randomization.

There were no intergroup differences for the aims of therapy, gross positions of participants, or for 10 of the 11 specific interventions. As expected, there were significantly (*P* < .001) more sessions involving splinting techniques for the SWIFT Cast (n = 313; 64.9%) than for the CPT group (n = 125; 31.4%) as a result of use of a SWIFT Cast. An AFO was used in more sessions (*P* = .005) for the CPT (n = 104; 26.1%) than for the SWIFT Cast group (n = 55; 11.4%). The number of individuals using an AFO in at least 1 CPT session was 9 of 51 (17.6%) of the SWIFT Cast and 19 of 54 (35.2%) of the CPT group (*P* = .002).

### Serious Adverse Events

No serious adverse events met the criteria for reporting to the National Research Ethics Service.

### Primary Outcome

At the primary time point (outcome), the mean (SD) walking speed (m/s) was 0.42 (0.37) for the CPT and 0.32 (0.34) for the SWIFT Cast group (Table 3). At follow-up, walking speed (m/s) had increased further to 0.53 (0.38) for the CPT and 0.43 (0.34) for the SWIFT Cast group. The intergroup difference was not significant at outcome (*P* = .345) or follow-up (*P* = .360).

**Table 3.** Walking Speed (m/s; primary outcome) at Outcome and Follow-up.

	CPT, Mean (SD) (Outcome, n = 45; Follow-up, n = 42)	SWIFT Cast, Mean (SD) (Outcome, n = 46; Follow-up, n = 36)	Effect Size (95% CI)	*P* Value	Adjusted Effect Size (95% CI)	*P* Value
Observed						
Outcome	0.42 (0.37)	0.32 (0.34)	−0.06 (−0.20,0.07)	.345	−0.06 (−0.19, 0.08)	.350
Follow-up	0.53 (0.38)	0.43 (0.34)	−0.08 (−0.23, 0.09)	.360	−0.08 (−0.24, 0.09)	.315
Imputed						
Outcome			−0.07 (−0.21, 0.08)	.369	−0.07 (−0.21, 0.07)	.340
Follow-up			−0.07 (−0.24, 0.11)	.458	−0.07 (−0.24, 0.11)	.430

Abbreviations: CPT, conventional physical therapy; SD, standard deviation; 95% CI, 95% confidence interval.

### Secondary Outcomes

At outcome (Tables 4 and 5), 65.1% of participants in the CPT and 61.3% of those in the SWIFT Cast group were able to walk 3 m independently compared with 24.1% and 19.6%, respectively, at baseline (Table 1). At follow-up, 75.7% of the CPT and 71.0% of the SWIFT Cast group were able to walk 3 m independently. The intergroup differences were not significant at outcome (*P* = .803) or at follow-up (*P* = .715).

**Table 4.** Secondary Measures at Outcome.^a^

	CPT (n = 45)	SWIFT Cast (n = 46)	Effect Size (95% CI)	*P* Value	Adjusted Effect Size (95% CI)	*P* Value
FAC	4.0 (3.0-4.0)	4.0 (2.0-4.0)		.822		
MRMI	33.93 (5.95)	32.65 (7.51)	−0.69 (−3.38, 1.96)	.610	−0.71 (−2.95, 1.58)	.539
Peak knee velocity, nonparetic (degrees/s)^b^	24.58 (36.40)	27.75 (54.62)	4.22 (−13.12, 23.11)	.647		
Peak knee velocity, paretic (degrees/s)^b^	21.93 (45.21)	19.60 (38.54)	−1.29 (−19.61, 14.81)	.884		
Able to walk	28/43^c^ (65.12)	27/44^c^ (61.36)	0.89 (0.37, 2.18)	.803		
Tibial angle to vertical at						
• Initial contact	−8.26 (6.13)	−6.83 (6.7)	1.10 (−2.10, 4.29)	.494		
• Foot flat	−1.16 (5.55)	−1.35 (4.87)	−0.55 (−3.35, 2.24)	.692		
• Midstance	8.54 (5.33)	7.83 (4.52)	−0.89 (−3.59, 1.81)	.509		
• Heel rise	27.42 (5.43)	26.22 (6.13)	−1.59 (−4.47, 1.28)	.272		
• Terminal contact	40.59 (8.34)	38.14 (8.77)	−2.62 (−6.93, 1.69)	.228		
• Midswing	21.08 (8.39)	20.68 (8)	−0.41 (−4.07, 3.24)	.821		
Ratio of stance times^d^	−0.17 (0.19)	−0.17 (0.16)		.506		
Ratio of step lengths^d^	−0.02 (0.05)	−0.03 (0.12)		.264		
Ratio peak angular velocities	0 (0.11)	−0.04 (0.18)		.943		

Abbreviations: CPT, conventional physical therapy; FAC, Functional Ambulation Category; MRMI, Modified Rivermead Mobility Index; SD, standard deviation; IQR, interquartile range.

a Values are mean (SD), median (IQR), or number (%).

b Bootstrap used because of nonnormality of residuals.

c Missing walking data for 2 participants.

d Based on Mann-Whitney test of absolute value treating those unable to walk as the largest values; mean (SD) refer only to those able to walk.

**Table 5.** Secondary Measures at Follow-up.^a^

	CPT, Mean (SD), n = 42	SWIFT Cast, Mean (SD), n = 36	Effect Size (95% CI)	*P* Value	Adjusted Effect Size (95% CI)	*P* Value
FAC	4.00 (4.00-5.00)	4.00 (4.00-5.00)		.257		
MRMI	36.14 (5.11)	35.74 (4.30)	−0.04 (−2.01,1.84)	.969	−0.16 (−1.95,1.66)	.860
Peak knee velocity, nonparetic (degrees/s)^b^	27.29 (41.62)	32.67 (51.89)	5.02 (−19.15,27.97)	.674		
Peak knee velocity, paretic (degrees/s)^b^	23.33 (34.34)	24.20 (45.62)	1.06 (−18.73,20.38)	.915		
Able to walk	28/37 (75.68)	22/31 (70.97)	0.82 (0.27,2.43)	.715		
Tibial angle at						
• Initial contact	−10.78 (4.91)	−8.55 (5.98)	2.45 (−0.41,5.31)	.092		
• Foot flat^b^	−3.59 (5.14)	−2.36 (4.64)	1.27 (−1.58,4.06)	.379		
• Midstance	6.48 (5.04)	8.2 (5.33)	1.97 (−1.13,5.06)	.208		
• Heel rise^b^	26.47 (6.12)	27.7 (6.18)	0.88 (−2.71,4.50)	.630		
• Terminal contact^b^	40.76 (8.19)	41.53 (8.45)	0.71 (−3.93,5.35)	.764		
• Midswing^b^	20.00 (8.74)	21.31 (5.67)	0.87 (−2.68,4.80)	.647		
Ratio of stance times^b^	−0.03 (0.07)	−0.04 (0.04)		.879		
Ratio of step lengths	−0.02 (0.14)	−0.00 (0.13)		.909		
Ratio of peak angular velocities	−0.11 (0.20)	−0.18 (0.16)		.499		

Abbreviations: CPT, conventional physical therapy; FAC, Functional Ambulation Category; MRMI, Modified Rivermead Mobility Index; SD, standard deviation; IQR, interquartile range.

a Values are mean (SD), median (IQR), or number (%).

b Bootstrap used because of nonnormality of residuals; mean (SD) refer only to those able to walk.

At outcome, median FAC scores had improved to 4.0 from a baseline of 1.0 in both groups. For MRMI, the improvements were from mean (SD) = 25.0 (8.4) to 33.95 (5.95) for the CPT and from 23.4 (7.5) to 32.65 (7.51) for the SWIFT Cast group. The intergroup differences were not statistically significant for either FAC (*P* = .822) or MRMI (*P* = .610). At follow-up, median FAC scores remained at 4.0 for both groups. Further improvement was observed for MRMI at follow-up when the mean (SD) score for the CPT was 36.14 (5.11) and for the SWIFT Cast group was 35.75 (4.30). The intergroup differences at follow-up were not significant for either FAC (*P* = .257) or MRMI (*P* = .969). There were no differences between groups for any other of the secondary outcomes at outcome or follow-up.

## Discussion

The results suggest that a SWIFT Cast used as an adjunct to CPT might not enhance walking recovery early after stroke. However, the use of AFOs in the CPT group was higher than expected from pretrial observations and discussion with clinical therapists. This potential confounder is discussed below. Considering the actual level of AFO use in the CPT group, the use of a SWIFT Cast early after stroke did not reduce recovery and, therefore, is possibly a clinical option for individuals early after stroke. Investigation of this possibility is warranted especially as a SWIFT Cast is different from an off-the-shelf or orthotist-made AFO. It is made from different materials, might have different properties, is custom-made by therapists who can readily adapt it during the rehabilitation period, and costs less (Condie et al^4^).

An obvious potential confounder in this trial is that significantly more people in the control group (35.2%) used an AFO during the CPT sessions than those in the SWIFT Cast group (17.6%). Awareness that AFO provision was part of routine CPT prompted the procedure by which clinical therapists agreed verbally to maintain their pretrial practice for the control group during the data collection period in consideration of the clinical uncertainty principle and genuine equipoise. Moreover, in keeping with best research practice and as stated in the ethical application and information given to participants, it was important to minimize the influence of research on routine clinical practice. It was anticipated from pretrial observation, conversations with clinical therapists, and research evidence^9^ that AFOs were used infrequently. To monitor the use of AFOs throughout the data collection period, the clinical and research therapists talked frequently. Despite these expectations and precautions, clinical provision of AFOs could be a confounding factor. Curiously, routine CPT also included use of a SWIFT Cast in 2.6% of the sessions provided for people in the control group, suggesting clerical error or that clinical therapists were “trying out” devices. In one clinical center, we were aware that the use of AFOs in CPT had probably increased and that this was related to production of some clinical guidelines.^10^ Interestingly, the strength of research evidence for the use of AFOs early after stroke did not increase from the pretrial situation. Indeed, the clinical guidelines recommendation used C-grade evidence for “where the aim of treatment is to have an immediate improvement on walking speed, efficiency or gait pattern or weight bearing during stance, patients should be assessed for suitability for an AFO by an appropriately qualified health professional.”^10^ Unfortunately, numerical data about AFO use during CPT sessions was only available to the trial team after hard-lock of the database. In both centers, it is possible that involvement in the trial increased clinical awareness of AFOs. With hindsight, it would have been beneficial to record use of AFOs in CPT before data collection began and to have applied to the ethical committee to enable us to undertake online monitoring of clinical practice and identify any variance from pretrial use of AFOs. Whether or not we would have been granted approval for such a request is unknown.

A systematic review identified only one previous randomized trial investigating the use of an AFO/cast in the first 3 months after stroke.^11^ Our systematic search did not identify any subsequent randomized trials. The earlier trial also found no difference in walking speed (effect size = 0.00 [−0.08, 0.08])^9,11^ but did report a highly significant increase (*P* = .0001) in FAC score from a median of 1 (1-1) to 2 (1-2).^12^ However, the earlier trial had a greater risk of potential bias than the one reported here for the following reasons: randomization was not to group but to the order of each individual using an AFO once the control walk (without AFO) had been made; observers were not masked to conditions because participants used the AFO during testing sessions; and an independent randomization service was not used. For the present trial, bias protection was also provided by adhering to the intention-to-treat principle and reporting all planned outcomes. This trial evaluated a highly visible SWIFT Cast; therefore, it was not possible to mask research therapists, participants, or clinical staff to the allocated intervention.

The 2 trials also differed in regard to the purpose of the devices and their use during outcome measures. The earlier trial evaluated the immediate effects on walking of an off-the-shelf plastic AFO to compensate for motor impairment,^12^ whereas the present trial evaluated the potential restorative effect on motor function of using an individually fitted SWIFT Cast over a 6-week period. It is possible that had measurements been collected while wearing a SWIFT Cast, walking improvement could have been detected. However, that was not the focus of the present trial because (*a*) it is already known that the immediate effects are positive both early and later after stroke^11^ and (*b*) the focus of rehabilitation in the first 3 months after stroke is to restore function rather than compensate for its loss.^13^

There is a possibility of differential response for subgroups of stroke survivors to the same intervention. For example, robotic gait training may be better than routine therapy for people early after stroke (mean 20 days) if they have low ability to voluntarily contract paretic muscle but not if they have high ability.^14^ This possibility is being tested through the indicators of response analysis of the embedded study of the present trial.^5^

That the present trial found no difference between the groups does not generalize to all types of AFOs. For example, stroke survivors walked further and negotiated stairs faster using a dynamic AFO than a carbon-composite AFO.^15^ It is possible, therefore, that an AFO custom-made by an orthotist for individuals early after stroke would restore motor function to a higher level than CPT alone. This hypothesis requires testing.

Another possible influence on these results is that insufficient walking training was undertaken to utilize better biomechanical alignment enabled by the SWIFT Cast. Intensity of training is known to be an important principle of neural plasticity,^16^ and there could be a critical threshold for efficacy.^17^ Both groups in this trial received essentially the same amount of CPT and walking training. Further investigation could determine the critical threshold for the number of stepping repetitions during walking wearing a SWIFT Cast.

The sample size for this trial was estimated by a power calculation informed by data from a group of similar stroke survivors receiving the same CPT as in this trial.^18^ However, the actual sample size was less than planned: 105 of 120 participants (87.5%). Published reports of earlier trials of lower-limb interventions early after stroke are scarce,^19^ which hinders direct comparison, but recruitment compares well with the finding that 78% of funded trials recruit to 80% of their target.^20^

An important strength of the trial reported here is that, on average, participants were recruited 20.8 days after stroke. Early rehabilitation is recommended because of potential for brain reorganization.^20^ In clinical practice, the majority of rehabilitation is provided in this time period, and yet most rehabilitation trials are conducted later in recovery.^19^

## Supplementary Material

Supplementary material
