# Supplementary material for: A Randomized Controlled Evaluation of the Efficacy of an Ankle-Foot Cast on Walking Recovery Early After Stroke: SWIFT Cast Trial
Source: Neurorehabil Neural Repair. 2016 Jan;30(1):40–8. doi: 10.1177/1545968315583724 (PMC4704299; doi:10.1177/1545968315583724)
Supplement: Supplementary material [file NNR583724_Supplementary_material.pdf]

Supplementary Appendix 1. Description of Treatment as Usual (Conventional Physical Therapy [CPT]).

All participants received treatment as usual in the form of CPT deemed appropriate for their presentation by the clinical physiotherapists and recorded using a standardised treatment schedule

(1). The treatment schedule consists of a recording form and explanatory manual describing the treatment and this has been used successfully with clinical physiotherapists in our recently completed trials comparing CPT and functional strength training (FST) for the lower limb (2). The clinical physiotherapists providing CPT were trained to use the treatment schedule and documented content and amount of treatment provided each day. The records of treatment provided for all participants were collected by a researcher each week.

The CPT as delivered in the centres for the present trial was a combination of hands-on techniques emphasising postural alignment and quality of movement together with goal-orientated task-specific training. Specific categories of physical therapy included: soft tissue mobilisation, facilitation of movement, splinting, resistive exercise, and functional mobility training. As in our earlier trial the purpose of the treatment schedule was not to constrain the clinical therapists in their choice of treatment for any individual (2-5). Clinical therapists were free to choose the techniques and the dose of treatment that they considered appropriate.

The Conventional Physical Therapy, Treatment as Usual, Intervention (1)

Aims

1. To reduce pain		3. To improve muscle activity/function		5. To improve gross mobility	
2. To improve sensory awareness		4. To improve postural control		6. To improve endurance	

Gross position of patient during activities used – what about kneeling postures?

1. Supine lying		4. non-paretic side lying		7. 4 pt kneeling		10. Standing	
2. Crook lying		5. Sitting - 90^0		8. 2 pt kneeling		11. Walking	
3. Paretic side lying		6. Sitting – perch		9. ½ kneeling		12. Other	

Equipment used

1. High hold/surface		4. Perching stool		7. Walking aid		10. Other	
2. Low hold/surface		5. Rolled up towel		8. Tilt table			
3. Hip high hold/surface		6. Gym ball		9. Standing frame			

Specific Physical Therapy interventions

1. Soft tissue mobilisation

1.1 Specific soft tissue mobilisation	
1.2 Passive movement	
1.3 Muscle stretching	

2. Facilitation of activity in specific muscles

2.1 Imagery of specific muscle activity	
2.2 Specific muscle activation	
2.3 Activation of muscle activity during function	

3. Facilitation of isolated (selective) joint movement

3.1 Imagery specific joint movement	
3.2 Active assisted isolated joint movement	
3.3 Facilitate specific joint movement during function	

4. Facilitation of co-ordinated (combined) movement

4.1. Imagery of co-ordinated patterns of movement	
4.2 Active assisted co-ordinated patterns of movement	
4.3 Facilitate co-ordinated movement during function	
4.4 Facilitate leg/foot activity from another body part	

5. Resistive exercise

5.1 Resistance from therapist	
5.2 Resistance from patient's bodyweight	
5.3 Resistance from equipment	

6. Specific sensory (tactile & proprioceptive) input

6.1 "Hands-on" techniques	
6.2 Provision of environmental surface	

7. Splinting techniques

7.1 Strapping	
7.2 Splinting	

8. Function – in lying towards sitting

8.1 PT "hands-on" techniques to re-ed posture	
8.2 Re-ed of funct act through specific mvmnt patterns	
8.3 Rolling – functional activity training	
8.4. Bridging - functional activity training	
8.5 Lying to sitting – functional activity training	
8.6 Sitting to lying - functional activity training	
8.7 Static sitting balance training	

9. Function – In sitting towards standing

9.1 PT "hands-on" techniques to re-ed posture	
9.2 Re-ed of funct act through specific mvmnt patterns	
9.3 Dynamic sitting balance training	
9.4 Transfers training	
9.5 Sit to standing – functional activity training	
9.6 Stand to sit – functional activity training	

10. Function – In standing towards walking

10.1 PT "hands-on" techniques to re-ed posture	
10.2 Re-ed of funct act through specific mvmnt patterns	
10.3 Static standing balance training	
10.4 Dynamic standing balance training	
10.5 One leg stand activities – functional training	

11. Function – Walking and onwards

11.1 PT "hands-on" techniques to re-ed posture	
11.2 Re-ed of funct act through specific mvmnt patterns	
11.3 Overground indoor walking training	
11.4 Overground outdoor walking training	
11.5 Treadmill walking/bicycle training	
11.6 Obstacle negotiation training	
11.7 Ascending/descending stair training	

Instructions for completion of recording form.

1. ONE FORM FOR EACH TREATMENT SESSION

   Please complete one form for each treatment session given to patients included as subjects in the Functional Strength Training lower limb clinical trial

2. TO COMPLETE THE AIMS SECTION

   Please place a tick in the box which best describes the aims relevant to the particular treatment session being recorded

3. TO COMPLETE THE GROSS POSITION SECTION

   Please place a tick in the box for every gross position used to deliver physiotherapy treatment during the treatment session being recorded

4. TO COMPLETE THE EQUIPMENT SECTION

   Please place a tick in the boxes which best describes the equipment used during the particular treatment session being recorded

5. TO COMPLETE THE SECTION "SPECIFIC PHYSICAL THERAPY INTERVENTIONS"

   Please place a tick in the boxes which best describe the treatment that was given to the patient during the particular treatment session being recorded.

6. FOR FURTHER DESCRIPTION OF ITEMS ON RECORDING FORM OVERLEAF

   Please refer to the accompanying document "Description of Lower Limb Treatment for Patients in FST Trial"

7. COMPLETED FORMS GIVEN TO RESEARCH TEAM

   When forms are complete please pass to Emma Cooke or Alison Lee (Researchers conducting the FST clinical trial)

Abbreviations for and glossary of terms used in recording form.

Act	Activity/activities
Environmental surface	A surface to enhance sensory input during functional activity e.g. sitting on a block of foam, walking on an exercise mat, walking on uneven ground
Facilitation	The application of an appropriate mode and dose (frequency, duration and intensity) of sensory stimulus provided by the therapist to access a desired active response from the patient
Funct	Function/functional
High hold/surface	A surface level with at least the mid-thoracic point of the patient to provide a hold and/or security during physical therapy intervention
Imagery	Mental rehearsal of a motor act that occurs in the absence of overt motor output
Low hold/surface	A surface level between the hip and mid-thoracic point of the patient to provide a hold and/or security during physical therapy intervention
Mvmnt	Movement
Physiotherapist	Person with professional Physiotherapy qualification
PT	Physical Therapy
Re-ed	Re-education
Rehabilitation Assistant	Person assisting the physiotherapist but who is not a qualified physiotherapist (e.g. student, nurse, technician, carer)

Supplementary Appendix 2. Description of SWIFT Cast Intervention.

Participants allocated to the experimental group received a soft-scotch ankle-foot cast (SWIFT Cast) in addition to CPT (6). A SWIFT Cast is a lightweight, semi-rigid cast extending from the metatarsal heads to the head of the fibula. It positions the paretic foot in relation to the shank so that plantarflexion and/or excessive pronation/supination of the foot is minimised during walking so that the ground reaction force vector assumes the normal direction i.e.: passing behind the knee at floor contact, through the knee in mid-stance and in front of the knee in terminal stance (7). It is made from Soft Cast and Scotch (3M PLC UK). The SWIFT Cast is lightweight (100-200g), semi-rigid and porous.

The SWIFT Cast was made on the first day of the intervention phase and fitted on the second day by researchers trained in the procedure before recruitment began. Training was delivered by a specialist physiotherapist with clinical expertise in the production and use of the SWIFT Cast. Competence in the technique was then assessed by a senior orthotics and prosthetics academic at the University of Strathclyde who was independent of the trial. Each researcher was required to be competent in the technique before making a SWIFT Cast for trial participants. Training updates were ongoing to maintain consistency in procedure and ensure quality throughout the trial and standardization across sites.

The SWIFT Cast was made with a participant in a supported sitting position that allowed hips, knees and ankles to be at 90^0. One researcher applied the materials whilst an assistant maintained the paretic ankle and foot in the plantargrade position, avoiding either pronation or supination at the subtalar joint. Details of making a SWIFT Cast were as set out below.

1. Ensure the following equipment is located at place of fitting:
    - Ground sheet

- Large basin
- Tube
- Blunt end scissors
- Different sizes of plaster boot
- Permanent marker pen
- 1 roll of Micropore
- 3 inch Stockinet
- 1 roll of Microfoam
- 1 roll of Leukotape
- 2 rolls of crepe bandage
- 2 rolls of 4 inch Soft Cast
- 2 rolls of 4 inch Scotch Cast
- Aprons
- Gloves
- Towels
- Basin of water at room temperature

2. All equipment set out on the floor near the fitter/assistant.
3. Fitter and assistant puts on aprons and gloves.
4. Participant’s lower leg exposed above knee.

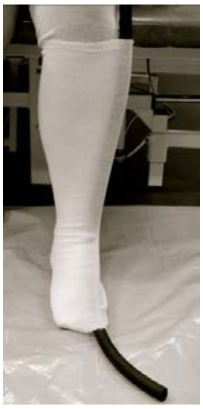

5. **Two** layers of stockinet fitted so that they extend a little above knee and beyond toes.
6. Tube inserted between layers at lateral border of foot [4th-5th mets] and lower leg.

7. Section of microfoam applied extending over malleloi.
8. Place foot on ground sheet so that the lower leg is inclined forwards by 5° - 10° to the vertical. Position of foot on ground sheet then marked with pen or micropore. The subtalar joint should be placed in an approximate neutral position. Inability to palpate the heads of the talus means the best way of achieving this is to keep the calcaneum in a vertical orientation.
9. Measure length of backslab. This will extend from the approximate level of the head of the fibula down the back of the leg, along dorsum of foot and a couple of inches beyond the toes. Measure this length with a tape measure and mark this on the ground sheet so that it can serve as a benchmark throughout the fitting.
10. Use this benchmark to roll out Scotch cast in layers. The material should be stretched gently and allowed to relax. Six layers should be built up, using more than one packet if required. This should then be left.
11. Open one packet of soft cast [use 4 inch width for large legs and 3 inch for standard or thin legs] and wrap around the leg working down from the head of fibula. Each strip should overlap by 50%. The ankle should be navigated with a figure of 8, as low as possible down lower leg. The “holder” should assist the wrapping by holding the lower leg [maintaining 5°-10 ° of inclination].

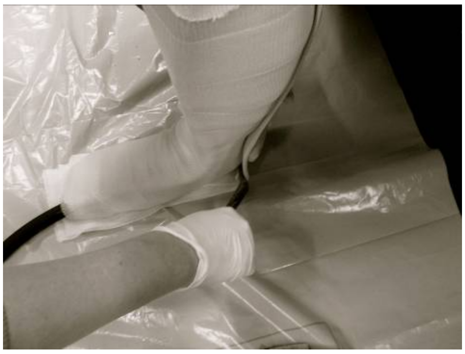

12. The backslab is then applied to the posterior calf and dorsum of foot.
13. The backslab is cut at the heel [low down and diagonally] to allow it to fold over [like hospital bed covers].

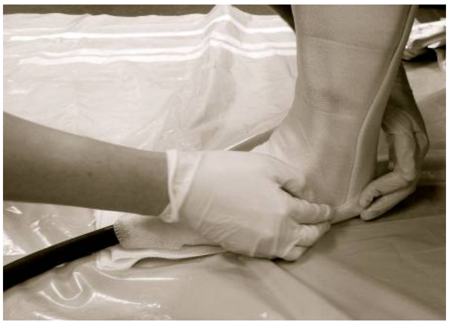

14. The backslab is then moulded with the hands to the calcaneum
15. The top layer of stockinet is rolled over the backslab at the knee to hold it in place.
16. The second roll of soft cast is then opened and "dunked" in the water and squeezed a little while in the water. This dampened roll is then wrapped around the leg in the same manner as the first soft cast (50% overlap).

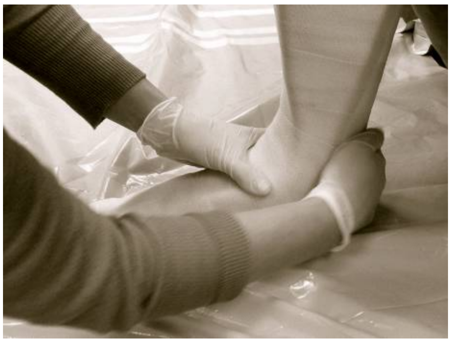

17. The cast should then be moulded to the shape of the leg by the fitter using their hands, paying particular attention to the heel [keeping a vertical orientation] and medial arch. During this process the 5°-10° of inclination should be maintained with the help of the handler.

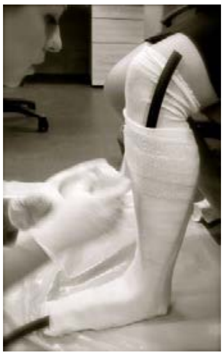
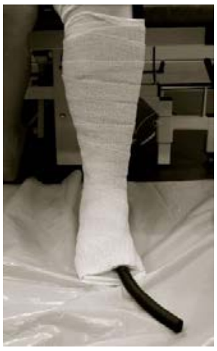

18. Wrap the wet crepe bandages around the whole cast and leave in place for at least 5 minutes.
19. Avoid movement at the ankle during this period as it may lead to creases.
20. Undo bandages

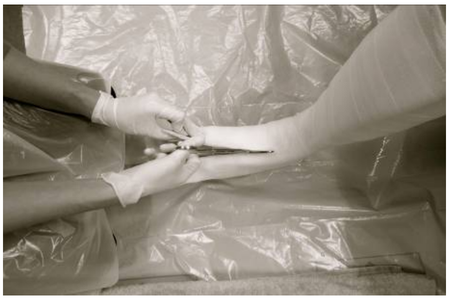

21. While the fitter is sitting at the side of the participant, a cut is made along the outside of the tube. It is easier to remove the tubing [by pulling] before cutting at the ankle.

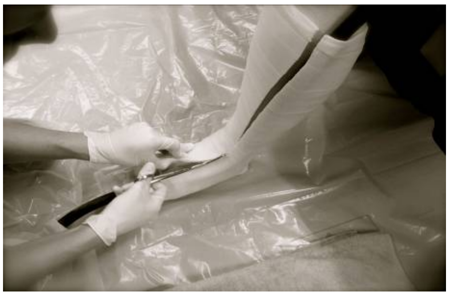

22. Before removing the cast, use the permanent marker to mark out the toe box. This should be big enough for the 5th toe to be exposed.
23. Remove cast.
24. Check skin for any markings.

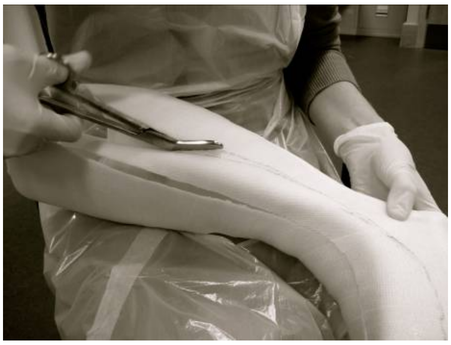

25. Cut out the anterior section of the cast, not extending beyond the malleoli or the head of fibula laterally so that the whole back and sides of the lower leg are covered.
26. Once the cast is trimmed, leave to set for 24 hours.

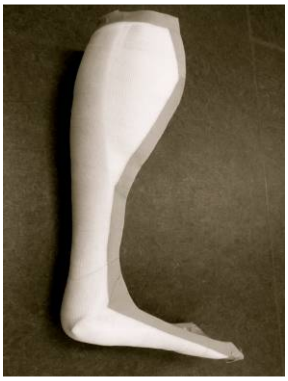

27. Twenty four hours later, trim any sharp corners and cover all edges with Leukotape tape.

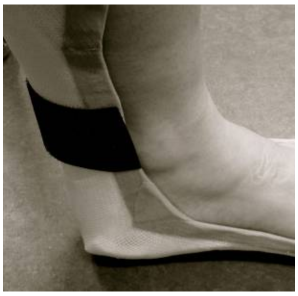

28. Apply two Velcro straps on the tibia section

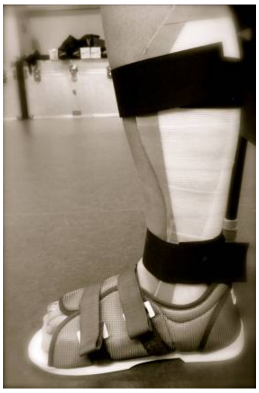

29. Select and fit correct size of plaster shoe

To ensure safe walking a strong plaster shoe (Darco Multifit Surgical Trauma Shoe rounded toe, Markell Shoe Co, USA) was fitted over the SWIFT Cast. To assess whether the SWIFT Cast enabled appropriate joint alignment the participant stood and stepped/walked forward whilst being observed in the sagittal plane. The observation checked that the paretic heel was on the floor at initial contact, there was no (or reduced) knee hyperextension, the tibia progressed smoothly forward during stance and the hip extended in terminal stance (i.e. minimizing gait abnormalities). If alignment was incorrect then a wedge was placed under the heel in the plaster shoe to tilt the tibia slightly forwards (tuning) and the observation assessment was repeated. The optimal forward tibia tilt aimed for was 8 degrees (8). A range of wedges was available to allow for the appropriate angle to be given to each participant so as to produce the optimal gait. Any pelvis asymmetry arising from leg length asymmetry was corrected using an insole in the shoe on the non-paretic foot.

During physical therapy sessions (CPT) the SWIFT Cast was worn for weight-bearing re-training of walking. As gait improved there were periods of walking re-training without wearing the SWIFT Cast aimed at re-education of lower limb movement control. All walking re-training interventions were drawn from the CPT treatment schedule (1) as deemed appropriate by clinical physiotherapists. Outside of physical therapy sessions participants were requested to wear the SWIFT Cast for the whole of their waking day initially. As gait improved the research therapist adjusted use of the SWIFT Cast as clinically appropriate. Each time the SWIFT Cast was applied/removed the lower limb was assessed for skin integrity (adverse event monitoring below). If a participant was discharged from an in-patient care setting during the six-week intervention period then he/she continued wearing the SWIFT Cast in their home. This was on the provision that the participant was regularly visited to ensure that skin integrity was monitored. If an individual regained a normal gait pattern when walking independently without the SWIFT Cast during the six-week intervention phase, then its use was discontinued as it was not clinically indicated. Use of a SWIFT Cast was also discontinued if a serious adverse occurred. If an individual discontinued using a SWIFT Cast before the end of the planned six-week intervention phase then the time period for which it was worn was recorded. Every effort was made to ensure that individuals who discontinued use of a SWIFT Cast participated in the outcome and follow-up measures (intention to treat principle).

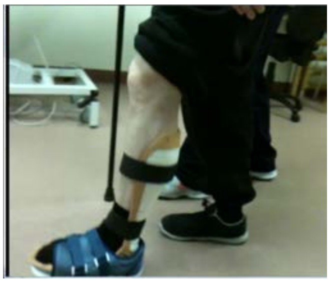

Using the SWIFT Cast during CPT

Supplementary Table 1. Conventional Physical Therapy (CPT) Received by Both Treatment Groups: Number of Sessions, Time Duration (Minutes) and Use of Therapy Staff.

	CPT (n=54)	SWIFT Cast (n=51)	p-value
Number of sessions			
Total	408	487	
Median (IQR) per participant	7 (0.12)	10 (0.16)	0.0952
Time duration (minutes)			
Median (IQR) total duration per participant	140 (0-350)	235 (0-590)	0.792
Mean (SD) duration per participant session	33.23 (15.54)	39.06 (16.44)	0.289[a]
Therapy staff use			
Median (IQR) total therapy time used per participant (hours)	2.84 (0.00-12.09)	5.34 (0.00-8.82)	
Mean (SD) therapy time used per session (hours)	0.89 (0.56)	0.98 (0.56)	0.916[a]
Median (IQR) number of physiotherapists used per session	1.0 (0-1)	1.0 (0-1)	0.227[a]
Median (IQR) number of assistants used per session	1.0 (0-1)	1.0 (0-1)	0.779[a]

IQR = interquartile range; SD = standard deviation; [a] = estimated from a random-effect linear regression model with patient as the random effect adjusting for factors to stratify the randomization – in order to account for the non-normality of the residuals a non-parametric bootstrap, with clustering variable as the participants, with 1,000 repetitions, was used to estimate the p-value and confidence interval.

Supplementary Table 2. The Aims of the Conventional Physical Therapy (CPT) Provided to the Two Treatment Groups.

Aim of session	Number of times the aims were recorded for sessions[a]		Odds ratio (95% CIs)	p-value
	CPT group (385 sessions)[b]	Swift Cast group (460 sessions)[c]		
Reduce pain	15 (3.9%)	12 (2.6%)	2.72 (0.34,21.75)	0.346
Improve sensory awareness	81 (21.0%)	65 (14.1%)	3.61 (0.74,17.58)	0.112
Improve muscle activity	300 (77.9%)	354 (77.0%)	1.04 (0.48,2.25)	0.929
Improve postural control	151 (39.2%)	177 (38.5%)	1.29 (0.59,2.82)	0.526
Improve gross mobility	320 (83.1%)	381 (82.8%)	0.84 (0.37,1.86)	0.660
Improve endurance	153 (39.7%)	144 (31.3%)	1.38 (0.54,3.52)	0.504

[a] 845 records with valid entries for aims of therapy; [b] = aims missing from records of 23 sessions; [c] = aims missing from records of 27 sessions.

Supplementary Table 3. The Positions Used in Conventional Physical Therapy (CPT) Sessions Provided to the Two Treatment Groups.

Aim of session	Number of times the positions were recorded for sessions[a]		Odds ratio (95% CIs)	p-value
	CPT group (n=408)	SWIFT Cast group (n=487)		
Supine lying	50 (13%)	66 (14.2%)	1.18 (0.59,2.34)	0.642
Crook lying	32 (8.3%)	43 (9.3%)	0.91 (0.39,2.12)	0.828
Paretic side lying	7 (1.8%)	2 (0.4%)	NA	0.087[b]
Non-paretic side lying	18 (4.7%)	18 (3.9%)	1.67 (0.51,5.44)	0.394
Sitting - 90 degrees	123 (32.0%)	179 (38.5%)	0.90 (0.51,1.59)	0.723
Sitting - perch	20 (5.2%)	25 (5.4%)	0.89 (0.31,2.61)	0.837
4-point kneeling	5 (1.3%)	2 (0.4%)	NA	0.254[a]
2-point kneeling	3 (0.8%)	1 (0.2%)	NA	0.334[a]
Half kneeling	2 (0.5%)	0	NA	0.204[a]
Standing	304 (79.2%)	361 (77.6%)	1.14 (0.69,1.89)	0.612
Walking	333 (86.7%)	404 (86.9%)	0.92 (0.49,1.72)	0.790
Other	10 (2.6%)	22 (4.7%)	0.45 (0.11,1.79)	0.256

[a] = 849 records with valid entries for positions used; [b] = Fisher's test used due to small numbers, this does not account for the non-independence of sessions; NA = not appropriate.

Supplementary Table 4. The Equipment Used in Conventional Physical Therapy (CPT) Sessions Provided for the Two Treatment Groups.

Aim of session	Number of times the equipment was recorded for sessions[a]		Odds ratio (95% CIs)	p-value
	CPT group (n=484)	SWIFT Cast group (n=403)		
High hold surface	58 (14.4%)	96 (20.5%)	0.66 (0.24,1.86)	0.434
Low hold surface	19 (4.7%)	35 (7.2%)	2.12 (0.22,20.43)[b]	0.517
Hip high hold surface	46 (11.4%)	100 (20.7%)	0.42 (0.16,1.08)	0.073
Perching stool	11 (2.7%)	20 (4.1%)	0.34 (0.05,2.60)	0.301
Rolled up towel	5 (1.2%)	9 (1.9%)	NA	0.592[c]
Gymball	7 (1.7%)	13 (2.7%)	0.42 (0.04,4.43)	0.472
Walking aid	288 (71.5%)	319 (65.9%)	1.12 (0.56,2.26)	0.749
Tilt table	0	0	NA	NA
Standing frame	5 (1.2%)	1 (0.2%)	NA	0.098[b]
Other	38 (9.5%)	58 (12.0%	0.83 (0.25,2.80)	0.766

[a] = 887 records with valid entries for equipment used; [b] = Functional Ambulation Categories data removed from model due to lack of fit;
[c] = Fisher's test used due to small numbers, this does not account for the non-independence of sessions; NA = not appropriate.

Supplementary Table 5. The Therapy Interventions Provided for Conventional Physical Therapy (CPT) Sessions for the Two Treatment Groups.

Therapy intervention	Number of times the intervention was recorded[a]		Odds ratio	p-value
	CPT group (n=398)	SWIFT Cast group (n=482)	(95% CIs)	
Soft tissue mobilisation	92 (23.1%)	118 (24.5%)	1.64 (0.52,5.15)	0.400
Activity in specific muscles	98 (24.6%)	166 (34.4%)	0.68 (0.25,1.82)	0.442
Isolated joint movement	58 (14.6%)	119 (24.7%)	0.53 (0.21,1.37)	0.193
Coordinated movement	104 (26.1%)	121 (25.1%)	1.23 (0.48,3.15)	0.671
Resistive exercise	82 (20.6%)	122 (25.3%)	0.83 (0.29,2.37)	0.722
Specific sensory input	67 (16.8%)	86 (17.8%)	1.19 (0.38,3.76)	0.768
Splinting techniques	125 (31.4%)	313 (64.9%)	0.06 (0.02,0.22)	**<0.001**
Strapping	5 (1.0%)	13 (3.3%)	6.53 (0.40,106.60)[b]	0.188
Ankle-foot orthosis	55 (11.4%)	104 (26.1%)	0.36 (1.99,53.95)	**0.005**
SWIFT Cast	264 (54.9%)	10 (2.6%)	0.00 (0.00,0.02)	**<0.001**
Function: lying to sitting	75 (18.8%)	75 (15.6%)	1.56 (0.80,3.06)	0.195
Function: sitting to standing	230 (57.8%)	270 (56.0%)	1.19 (0.61,2.29)	0.612
Function: standing to walking	249 (62.6%)	303 (62.9%)	1.12 (0.62,2.01)	0.706
Function: walking & onwards	354 (88.9%)	435 (90.3%)	0.80 (0.39,1.63)	0.543

[a] = 880 records with valid entries for therapy intervention; [b] = Functional Ambulation Categories removed from model because of poor fit

Supplementary Table 6. Comparison of Secondary Measures at Outcome. Imputed results. Values are mean (SD) or n(%).

	Effect size (95% CI)	p-value	Adjusted Effect size (95% CI)	p-value
FAC		0.778		
MRMI	-0.85 (-3.55,1.85)	0.532	-0.56 (-2.97,1.84)	0.641
Peak knee velocity, non-paretic degs/s	-0.51 (-38.93,37.90)	0.978		
Peak knee velocity, paretic degs/s	-2.51 (-22.49,17.47)	0.803		
Able to walk	0.77 (0.31,1.86)	0.562		
Tibial angle at:				
Initial contact (TAIC)	-1.05 (-7.31,5.21)	0.727		
Foot flat (TAFF)	-1.29 (-6.49,3.92)	0.610		
Mid-stance (TAMS)	-0.28 (-5.24,4.67)	0.905		
Heel rise (TAHR)	-0.63 (-4.51,3.25)	0.744		
Terminal contact (TATC)	-1.13 (-7.70,5.43)	0.723		
Mid-swing (TASW)	0.09 (-6.02,6.19)	0.977		
Ratio of stance times (RST) *		0.682		
Ratio of step lengths (RSL) [#]		0.396		
Ratio peak angular velocities (RPAV)		0.462		

FAC = Functional Ambulation Category, MRMI = Modified Rivermead Mobility Index; degs/s = degrees per second;
[*] bootstrap used due to non-normality of residuals; mean (SD) refer only to those able to walk.

Supplementary Table 7. Comparison of Secondary Measures at Follow-up. Imputed results. Values are mean (SD) or n(%).

	Effect size (95% CI)	p-value	Adjusted Effect size (95% CI)	p-value
FAC		0.264		
MRMI	-0.50 (-2.57,1.57)	0.633	-0.34 (-2.30,1.62)	0.729
Peak knee velocity, non-paretic degs/s	8.92 (-15.25,33.09)	0.461		
Peak knee velocity, paretic degs/s	-3.88 (-27.28,19.52)	0.741		
Able to walk	0.77 (0.29,2.04)	0.604		
Tibial angle to vertical at:				
Initial contact (TAIC)	0.01 (-7.56,7.58)	0.997		
Foot flat (TAFF)	-0.47 (-8.32,7.37)	0.899		
Mid-stance (TAMS)	1.83 (-4.98,8.64)	0.580		
Heel rise (TAHR)	1.34 (-7.92,10.60)	0.750		
Terminal contact (TATC)	1.68 (-6.39,9.76)	0.671		
Mid-swing (TASW)	0.38 (-7.70,8.46)	0.923		
Ratio of stance times (RST) *		0.557		
Ratio of step lengths (RSL) #		0.773		
Ratio peak angular velocities (RPAV)		0.510		

FAC = Functional Ambulation Category, MRMI = Modified Rivermead Mobility Index; degs/s = degrees per second;
* bootstrap used due to non-normality of residuals; mean (SD) refer only to those able to walk

Supplementary References

1. Pomeroy VM, Cooke E, Hamilton S, Whittet A, Tallis RC. Development of a Schedule of Current Physiotherapy Treatment Used to Improve Movement Control and Functional Use of the Lower Limb after Stroke: A Precursor to a Clinical Trial. Neurorehabil Neural Repair [Internet]. 2005;19:350–9. Available from: http://nnr.sagepub.com/content/19/4/350.abstract
2. Cooke E V, Tallis RC, Clark A, Pomeroy VM. Efficacy of Functional Strength Training on Restoration of Lower-Limb Motor Function Early After Stroke: Phase I Randomized Controlled Trial. Neurorehabil Neural Repair 24:88–96.
3. Donaldson C, Tallis R, Miller S, Sunderland A, Lemon R, Pomeroy V. Effects of Conventional Physical Therapy and Functional Strength Training on Upper Limb Motor Recovery After Stroke: A Randomized Phase II Study. Neurorehabil Neural Repair [Internet]. 2009;23:389–97. Available from: http://nnr.sagepub.com/cgi/content/abstract/23/4/389
4. Hunter SM, Hammett L, Ball S, Smith N, Anderson C, Clark A, et al. Dose–Response Study of Mobilisation and Tactile Stimulation Therapy for the Upper Extremity Early After Stroke. Neurorehabil Neural Repair [Internet]. 2011;25:314–22. Available from: http://nnr.sagepub.com/cgi/content/abstract/25/4/314
5. Cowles T, Clark A, Mares K, Peryer G, Stuck R, Pomeroy V. Observation-to-Imitate Plus Practice Could Add Little to Physical Therapy Benefits Within 31 Days of Stroke: Translational Randomized Controlled Trial. Neurorehabil Neural Repair [Internet]. 2013;27:173–82. Available from: http://nnr.sagepub.com/cgi/content/abstract/27/2/173
6. Edwards S, Charlton PT. Splinting and the use of orthoses in the management of patients with neurological disorders. Neurological Physiotherapy (second edition). Churchill Livingstone; 2002. p. 219–53.

7. Kirtley C. Clinical gait analysis: theory and practice. Chrurchill Livingstone; 2006.
8. Owen E. The importance of being earnest about shank and thigh kinematics especially when using ankle-foot orthoses. Prosthet Orthot Int. 2010;34:254–69.
